# A spatially distributed rainfall dataset for West Java, Indonesia

**DOI:** 10.1016/j.dib.2025.111974

**Published:** 2025-08-12

**Authors:** Dwi Yoga Primartono, Rahmat Hidayat, Supari, Rakhmat Prasetia, Muh Taufik

**Affiliations:** a Department of Geophysics and Meteorology, Kampus IPB Darmaga, IPB University, Bogor, 16680, Indonesia; b Climatological Station West Java, BMKG, Bogor, 16680, Indonesia; c Centre for Climate Change Information, Indonesia Agency for Meteorology Climatology and Geophysics (BMKG), Jakarta, 10610, Indonesia; d Centre for Environmental Research, Kampus IPB Dramaga, IPB University, Bogor, 16680, Indonesia

**Keywords:** Climatology, Interpolation, Inverse distance weighting, Rain gauge, Topography

## Abstract

Rainfall data availability is a basis of climate analysis and application, but its spatial distribution based on observed rainfall at local scale remains a research challenge. A spatially distributed rainfall at a finer resolution is the foundation for coping uncertain climate change and water resource planning and management. Here, we established a daily grid dataset for observed rainfall of West Java, Indonesia. The data were *from* 1991-2020 at daily resolution from 162 rain gauges covering various terrains and climate zone, which were monitored by *the Indonesian Agency for Meteorology Climatology and Geophysics* (BMKG). We used the inverse distance weighting (IDW) approach to spatially interpolate rainfall at *0.05^0^* grid resolution. In addition, timeseries of monthly and annual rainfall were generated from the daily dataset. Further, the spatial rainfall data *is* useful for identifying local climate, adaptation strategy for hydro-meteorological hazard, and water resource planning.

Specifications Table

**Table:** 

Subject	Earth & Environmental Sciences
Specific subject area	Interdisciplinary fields include hydrology, climatology, applied meteorology, and environmental science
Type of data	Table, GeoTIFF, Chart, Graph
Data collection	A total of 162 rain gauges across topography and climate zones of West Java (Table 3) were used for the input data. In each gauge, rainfall was daily monitored at 07:00 AM local time. The distribution of rain gauges is presented in Figure 1. Missing gap of the rainfall records is obvious, and we addressed it by developing the interpolated gridded rainfall data *from* 1991-2020.
Data source location	Country: Indonesia Province: West Java Institution: Indonesia Agency for Meteorology Climatology (BMKG); Climatological Station of West Java. West Java is located at 106° – 108° E and 6° – 8° S
Data accessibility	Repository name: Mendeley Data Data identification number: 10.17632/7swpy42ngm.3 Direct URL to data: https://data.mendeley.com/datasets/7swpy42ngm/3

## 1 Value of the Data

• By integrating local rainfall observations, the dataset provides more reliable spatial representations compared to coarser global datasets, improving the detection of local climatic variability and supporting more accurate climate modelling.
• The interpolated rainfall dataset serves as a valuable reference for spatial rainfall distribution modeling, particularly in areas with limited observational coverage, offering critical insights for improving hydrological and climatological research in West Java.
• The dataset supports investigations into the influence of topographic variation on rainfall distribution, contributing to more effective regional water resource management strategies.
• This dataset aids in evaluating climate variability impacts, particularly *El Niño-Southern Oscillation (ENSO)-*induced fluctuations, in one of Indonesia’s most agriculturally and economically vital provinces.
• Accurate rainfall datasets are useful for planning and decision-making in agriculture, disaster preparedness, and managing water resource, allowing accurate hydrometeorological assessments of drought based on local seasonal zones in West Java.

## 2 Background

Rainfall data availability is crucial for understanding climate patterns [1], assessing extreme hydrometeorological events [2], and formulating effective mitigation strategies for hydrometeorological hazards [3]. But its spatial distribution based *on rain gauge observation* is challenging especially in the tropical region of Indonesia, *where topography and atmospheric conditions influence rainfall variability. In coastal area, land-see breeze circulation dominates diurnal variability of rainfall* [4], *while in the western mountainous region topography drives rainfall distribution* [5,6]*.* In addition, missing data is a persistent challenge for data monitoring, affecting the reliability of climatological and hydrological analyses [7]. Here, we address these gaps by using interpolation techniques to estimate rainfall in unsampled locations. The Inverse Distance Weighting (IDW) is widely applied due to its computational efficiency and capacity to estimate rainfall values based on the proximity of observational points [8].

**Fig. 1:**
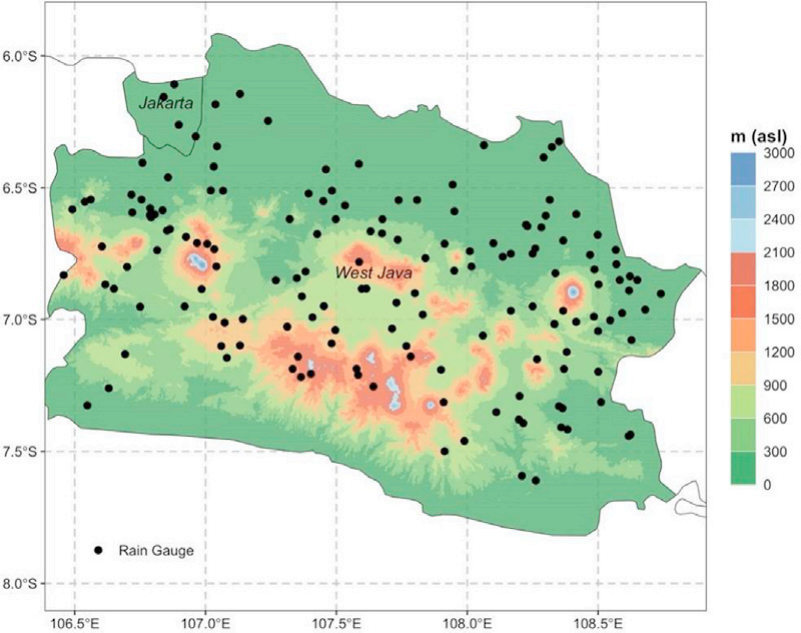
Rain gauges (black dots) distributions at West Java and Jakarta with total of 162 stations overlays *on a topography map.*

This study presents a comprehensive dataset on the spatial distribution of rainfall stations and the extent of missing data from 162 monitoring stations across West Java (Fig. 1)*,* over the period 1991–2020. Further, annual rainfall interpolation using the IDW method is performed to generate a spatial representation of rainfall distribution across the region. *The dataset is designed to support advanced research on rainfall variability and its implications* for water resource management in West Java.

## 3 Data Description

We provide a description on the linked repository of a daily rainfall for 1 January 1991 – 31 December 2020 in West Java, Indonesia, at a spatial resolution of 0.05^0^. *The data was interpolated from 162 rain located across a wide range of elevation* (Table 1).

**Table 1:** Distribution of gauge stations and their mean annual rainfall following elevation gradient in West Java

Elevation (m)	Area coverage (%)	Number of rain gauges	Number of grids	Mean Annual Rainfall (mm)
0 – 500	65.1	104	808	2,679
500 – 1000	23.0	40	285	2,819
1000 – 1500	8.2	13	102	2,811
1500 – 2000	3.3	5	41	2,656
2000 – 3000	0.4	-	5	2,935
Total	100%	162	1241	

Lowland area (<500 m) is the most dominant elevation (65%, Table 1) in the study site, in which it correspondingly contributes to almost two third of rain gauges (104 of 162). 40 gauges is located at elevation of 500 – 1000 m. Further, 18 gauges represents highland area (>1000 m), but there is no monitoring site above 2000 m.

The dataset we provide is in txt format that comprises of daily information such as: date, coordinates, and magnitude of rainfall for each grid of 0.05o. Table 2 provides the structure of datasets in wide format, which comprises of x-coordinate, y-coordinate, and series of daily rainfall according to the date. To offer more choice on the dataset types, we provide a dataset in GeoTIFF format that is easily handle using R language or Phyton software. The rainfall datasets at monthly and annual scale are provided for both txt and GeoTIFF format types with similar data structure.

**Table 2:** The structure of dataset in txt format containing daily rainfall for each grid

X	y	X1991.01.01	X1991.01.02	…	X2020.12.31
107.05	-5.95	4.47	4.25	…	10.57
107.1	-5.95	5.02	1.91	…	9.01
…					
108.45	-7.8	17.74	20.04	…	25.98

**Fig. 2:**
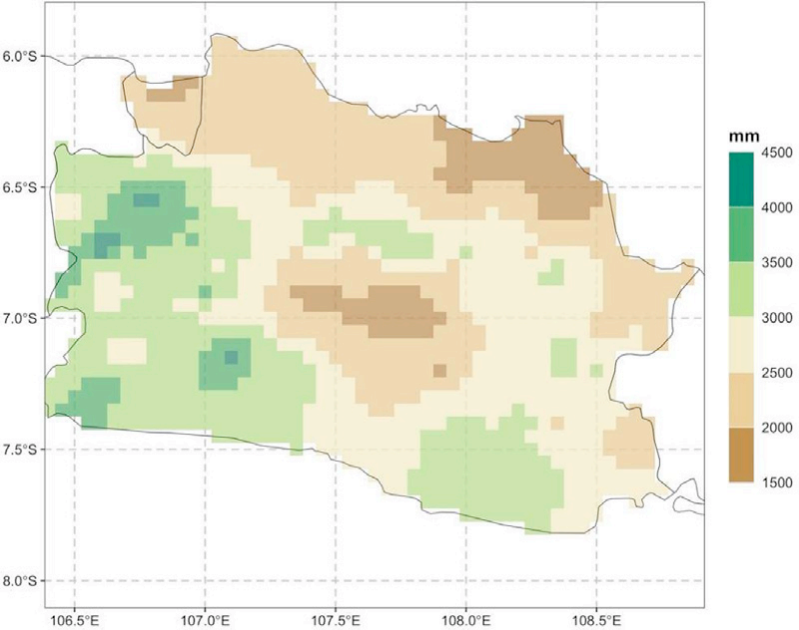
Mean annual rainfall across West Java for 1991-2020

The mean annual rainfall ranges from 1591 to 4,455 (Fig. 2), representing lowland and highland variability. A relatively low rainfall is found in the northern coastal of West Java and in the basin of Citarum, which located in the heart of West Java.

## 4 Experimental Design, Materials and Methods

Daily rainfall data were interpolated using observations from station-level rain gauges across West Java, Indonesia. A total of 162 rain gauge stations were distributed across varying elevation. The interpolation process was relied on all available data from all stations, which varying data gaps (Table 3). This incompleteness data has challenged for the interpolation for 30-year period (10,958 days) covering the West Java [9].

**Table 3:** List of stations and their data gaps

No	ID	Latitude (N/S)	Longitude (E/W)	Elev (m)	Missing Data (%)
1	32010102a	6.4604°S	106.8564°E	122	1.12
2	32010606a	6.5100°S	107.0200°E	276	8.61
3	32010704a	6.4198°S	107.0313°E	60	6.96
4	32011202a	6.5445°S	106.7553°E	177	10.99
5	32011401a	6.7226°S	106.6039°E	936	7.53
6	32011504a	6.5934°S	106.7199°E	219	3.08
7	32012202a	6.5528°S	106.5386°E	373	2.37
8	32012401a	6.6624°S	106.8529°E	488	7.33
9	32012508a	6.6862°S	106.9253°E	882	2.14
10	32012511a	6.7092°S	106.9674°E	1139	0.08
11	32012602a	6.6569°S	106.8638°E	495	2.62
12	32012802a	6.7368°S	106.8151°E	471	22.91
13	32012902a	6.6039°S	106.7873°E	247	13.72
14	32013301a	6.4053°S	106.7589°E	96	3.64
15	32013401a	6.5259°S	106.7166°E	146	25.55
16	32020102a	6.5818°S	106.4909°E	501	2.78
17	32020402a	6.8844°S	106.9851°E	863	9.72
18	32020503a	6.8314°S	106.4577°E	632	5.04
19	32020603a	6.8830°S	106.6500°E	563	22.53
20	32020701a	7.1310°S	106.6924°E	595	1.96
21	32020903a	6.9516°S	106.7501°E	301	13.83
22	32021501a	6.8000°S	106.7000°E	643	6.94
23	32021502a	6.8670°S	106.6167°E	512	4.46
24	32022103a	7.2600°S	106.6300°E	313	8.08
25	32022402a	7.3256°S	106.5481°E	129	8.64
26	32023203a	6.5442°S	106.5612°E	413	22.24
27	32024001a	6.9900°S	107.0284°E	708	4.45
28	32030601a	6.8511°S	107.2687°E	294	36.33
29	32031001a	6.7127°S	107.0063°E	1264	1.41
30	32031102a	6.7985°S	107.0414°E	1166	12.75
31	32031401a	7.1446°S	107.0815°E	759	1.64
32	32031402a	7.1000°S	107.0600°E	965	1.4
33	32031403a	7.0978°S	107.1317°E	872	33.36
34	32031501a	7.0115°S	107.0730°E	984	17.24
35	32031504a	6.9983°S	107.1423°E	988	21.48
36	32032805a	6.7328°S	107.0333°E	1131	39.18
37	32041504a	7.2100°S	107.5833°E	1545	4.74
38	32041510a	7.1864°S	107.5771°E	1433	6.95
39	32042501a	6.9813°S	107.8301°E	688	6.97
40	32042901a	7.0338°S	107.7129°E	680	1.12
41	32043110a	7.2537°S	107.6414°E	1634	1.68
42	32043502a	7.1000°S	107.7676°E	901	8.58
43	32043601a	7.1398°S	107.7841°E	1492	40.04
44	32043701a	7.0394°S	107.4959°E	805	0.83
45	32043801a	7.2057°S	107.4028°E	1755	0.28
46	32043902a	7.2178°S	107.3647°E	1540	25.58
47	32043903a	7.1404°S	107.3533°E	1494	19.24
48	32043908a	7.0903°S	107.4817°E	1036	3.61
49	32043909a	7.1869°S	107.3329°E	1323	0.84
50	32050401a	7.1900°S	107.9000°E	713	18.32
51	32051401a	7.0604°S	108.0600°E	643	32.56
52	32051901a	7.3133°S	107.9100°E	1088	1.5
53	32052401a	7.4996°S	107.9134°E	676	33.09
54	32060205a	7.5929°S	108.2090°E	331	11.98
55	32060501a	7.6103°S	108.2620°E	256	23.14
56	32061302a	7.4604°S	107.9892°E	857	23.72
57	32062001a	7.4167°S	108.3833°E	314	17.57
58	32062002a	7.4087°S	108.3590°E	306	36.46
59	32062402a	7.3509°S	108.1111°E	431	31.65
60	32070101a	7.3285°S	108.3506°E	212	40.87
61	32070104a	7.3362°S	108.3643°E	198	15.05
62	32070801a	7.1500°S	108.2667°E	786	12.54
63	32070901a	7.1870°S	108.3698°E	384	6.73
64	32071001a	7.1225°S	108.3803°E	682	28.9
65	32071502a	7.1974°S	108.5007°E	347	17.98
66	32071702a	7.4415°S	108.6170°E	17	26.65
67	32073002a	7.3128°S	108.5115°E	130	27.23
68	32073501a	7.4364°S	108.6239°E	44	7.78
69	32080201a	7.0429°S	108.5001°E	263	14.46
70	32080401a	7.0770°S	108.6276°E	165	28.17
71	32080801a	7.0027°S	108.5464°E	245	7.49
72	32080901a	6.9890°S	108.4843°E	467	18.07
73	32081002a	6.9755°S	108.5920°E	195	14.46
74	32081101a	6.9624°S	108.6802°E	29	9.68
75	32081301a	6.8669°S	108.5022°E	413	4.98
76	32081402a	6.8097°S	108.4863°E	312	6.42
77	32081704a	7.0086°S	108.4163°E	726	8.06
78	32090201a	6.9020°S	108.7400°E	18	6
79	32090601a	6.8899°S	108.6187°E	35	11.43
80	32090602a	6.8500°S	108.6500°E	15	28.42
81	32090702a	6.8500°S	108.5830°E	145	9.72
82	32091203a	6.7900°S	108.5700°E	30	9.17
83	32091401a	6.7356°S	108.5677°E	0	10.63
84	32091901a	6.7545°S	108.4695°E	48	4.51
85	32091902a	6.6786°S	108.4987°E	8	13.15
86	32092207a	6.8358°S	108.6217°E	28	9.99
87	32092601a	6.7000°S	108.3670°E	34	29.34
88	32092801a	6.6000°S	108.4167°E	5	2.3
89	32100101a	6.9667°S	108.1667°E	539	1.68
90	32100301a	7.0167°S	108.3333°E	577	14.99
91	32100302a	7.0167°S	108.3333°E	577	2.52
92	32100401a	6.9500°S	108.2500°E	459	4.18
93	32100402a	6.9667°S	108.3667°E	1016	1.41
94	32100801a	6.6403°S	108.2247°E	20	2.79
95	32100901a	6.8241°S	108.3371°E	455	10.56
96	32101102a	6.7500°S	108.2500°E	52	4.16
97	32101106a	6.7300°S	108.2600°E	45	6.05
98	32101301a	6.7500°S	108.1667°E	35	5.89
99	32101502a	6.6454°S	108.2300°E	24	30.08
100	32101504a	6.6500°S	108.2833°E	23	5.29
101	32110301a	6.7617°S	108.1358°E	45	18.61
102	32110701a	6.7405°S	108.0099°E	358	15.71
103	32110801a	6.7985°S	108.0163°E	436	26.73
104	32111001a	6.7122°S	107.9137°E	315	28.92
105	32111101a	6.9000°S	107.8000°E	864	17.72
106	32112101a	6.7668°S	107.8404°E	562	6.03
107	32112201a	6.8145°S	107.9504°E	547	1.1
108	32112401a	6.7617°S	108.1358°E	45	46.57
109	32112501a	6.7100°S	108.1000°E	48	0.57
110	32120102a	6.4878°S	107.9444°E	29	5.77
111	32120103a	6.5891°S	107.9511°E	58	0.9
112	32120201a	6.3389°S	108.0639°E	2	4.51
113	32120304a	6.3851°S	108.2921°E	2	4.34
114	32120601a	6.5456°S	108.3154°E	17	3.83
115	32120801a	6.3248°S	108.3510°E	1	3.85
116	32120802a	6.6057°S	108.3001°E	22	0.84
117	32121502a	6.3457°S	108.3237°E	5	2.24
118	32130102a	6.6731°S	107.6744°E	564	9.74
119	32130104a	6.6192°S	107.6760°E	308	0.9
120	32130301a	6.5469°S	107.7378°E	98	5.01
121	32130502a	6.4099°S	107.5860°E	31	22.79
122	32130703a	6.5462°S	107.8084°E	66	15.72
123	32132302a	6.6648°S	107.6306°E	534	11.75
124	32132601a	6.6969°S	107.7344°E	468	9.68
125	32140101a	6.5500°S	107.4500°E	83	0
126	32140102a	6.5107°S	107.4830°E	77	10.56
127	32140201a	6.4300°S	107.4600°E	55	1.33
128	32140205a	6.5667°S	107.5330°E	124	0
129	32140302a	6.6754°S	107.4267°E	496	0.84
130	32140303a	6.5215°S	107.3938°E	122	1.68
131	32140305f	6.6189°S	107.3215°E	250	20.29
132	32141601a	6.6188°S	107.4978°E	292	0
133	32160901a	6.1442°S	107.1316°E	3	2.49
134	32161801a	6.3431°S	107.0438°E	39	7.05
135	32162003a	6.2465°S	107.2387°E	11	12.06
136	32162202a	6.5111°S	107.0671°E	181	6.41
137	32170201a	6.7816°S	107.5870°E	1547	9.48
138	32170702a	6.8175°S	107.3821°E	353	9.48
139	32170706a	6.8427°S	107.3488°E	321	7.14
140	32170707a	6.9123°S	107.3681°E	648	3.08
141	32171101a	6.9490°S	107.4522°E	653	3.33
142	32171402a	6.9917°S	107.4087°E	710	14.79
143	32171502a	7.0263°S	107.3119°E	1025	19.42
144	32171503a	7.0271°S	107.3121°E	1028	8.94
145	32710101a	6.6117°S	106.7914°E	266	2.25
146	32710201a	6.6006°S	106.8058°E	268	1.24
147	32710302a	6.5773°S	106.7882°E	226	1.47
148	32710401a	6.5850°S	106.8358°E	224	8.08
149	32710501a	6.5988°S	106.7956°E	269	4.18
150	32720601a	6.9500°S	106.9200°E	491	14.75
151	32730201a	6.8826°S	107.6144°E	794	0.84
152	32730701a	6.8835°S	107.5968°E	798	2.17
153	32732602a	6.9360°S	107.7295°E	685	3.62
154	32750201a	6.1837°S	107.0378°E	12	18.85
155	32750901a	6.3056°S	106.9623°E	36	15.89
156	32780401a	7.2900°S	108.2000°E	393	7.77
157	32780502a	7.3791°S	108.1974°E	314	29.76
158	32781001a	7.3929°S	108.2143°E	362	29.95
159	DKI2	6.1556°S	106.8400°E	3	12.39
160	DKI44	6.1556°S	106.8400°E	3	1.7
161	DKI45	6.1078°S	106.8805°E	2	1.34
162	DKI8	6.2616°S	106.8976°E	26	33.45

Rainfall data was obtained from Indonesia Agency for Meteorology Climatology and Geophysics (BMKG), which was in MS Excel format, and we collated them into R using readxl package [10]. We did the gross error check and advance quality control of rainfall data based on procedures developed in BMKG [11].

Then, the data were tidied up using the tidyverse package [12] to form long format data, in which each row consisted of geographical coordinate (longitude, latitude), daily rainfall, and date information. Further, we employed the inverse distance weighting (IDW) interpolation technique to interpolate daily rainfall over a grid that covered 106.1° – 109° E and 8° – 5.8 °S, with a spatial resolution of 0.05°. The IDW formula is:

$$\overset{⌢}{Z}\left(s_{0}\right)=\frac{\sum_{i=1}^{N}w_{i}Z\left(s_{i}\right)}{\sum_{i=1}^{N}w_{i}}$$

where $w_{i}=\frac{1}{d_{i}^{p}}$ represents the weight assigned to each observation Z (s_i_), based on its distance d*i* from the interpolation point s_0_, with power parameter p = 2. During the interpolation process, the estimation of rainfall depends on the 10 nearby stations.

We developed R code to generate the spatial datasets based on terra package [13]. The R code is available at https://github.com/mathiidayats/idwR

## Limitations

Not applicable.

## Ethics Statement

This work does not involve human subjects and animal experiments.

## Data Availability

Mendeley DataA Spatial Rainfall of West Java (Original data)
